# Validation of the PD home diary for assessment of motor fluctuations in advanced Parkinson’s disease

**DOI:** 10.1038/s41531-022-00331-w

**Published:** 2022-06-02

**Authors:** Matthias Löhle, Alexander Bremer, Florin Gandor, Jonathan Timpka, Per Odin, Georg Ebersbach, Alexander Storch

**Affiliations:** 1 Department of Neurology, University Medical Center Rostock, Rostock, Germany; 2 German Center for Neurodegenerative Diseases (DZNE) Rostock-Greifswald, Rostock, Germany; 3 Movement Disorders Clinic, Beelitz-Heilstätten, Beelitz, Germany; 4 Department of Neurology, Otto-von-Guericke University, Magdeburg, Germany; 5 Division of Neurology, Department of Clinical Sciences Lund, Lund University, Lund, Sweden; 6 Department of Neurology, Skåne University Hospital, Lund, Sweden

**Keywords:** Parkinson's disease, Parkinson's disease

## Abstract

The Parkinson’s disease (PD) home diary is frequently used in clinical trials to measure efficacy of medical treatments for motor fluctuations in advanced PD. This prospective study in fluctuating PD patients examines the validity of the diary for quantification of motor states in comparison to direct clinical observation. 51 patients (median age: 65 years, disease duration: 11 years) completed the diary half-hourly for two consecutive days and were simultaneously rated by an experienced observer, who independently evaluated motor states half-hourly throughout daytime. Overall agreement (Cohen’s kappa) between patient and observer diary entries was 59.8% (0.387). Patients documented more On without dyskinesia (52.3% vs. 38.9%, *P* < 0.001) and less On with dyskinesia (21.5% vs. 34.2%, *P* < 0.001), whereas proportions for Off intervals were not different between patient and observer diaries (26.2% vs. 27.0%, *P* = 0.97). Temporal agreement between diary ratings was unsatisfactory, particularly for On with dyskinesia. Taken together, our study suggests that the PD home diary only inadequately reflects actual motor states compared to direct clinical observation.

## Introduction

Advanced stages of Parkinson’s disease (PD) are often characterized by the presence of motor fluctuations, which affect about 50% of patients after 5 years of disease^1^. Moreover, PD is accompanied by a range of non-motor symptoms that further complicate management of later stages of the disease, which could thus also be referred to as “complex phase PD”^2^.

In 2000 and subsequent years, Hauser et al. developed the PD home diary to quantify motor fluctuations as patient-defined outcome measure for clinical trials^3–5^. In this diary, which is nowadays often also referred to as the Hauser diary, patients are asked to indicate their predominant status during half-hour time periods throughout the day using the categories Asleep, Off, On without dyskinesias, On with non-troublesome dyskinesia, and On with troublesome dyskinesia. The diary allows calculation of daily Off-time and daily On-time with and without troublesome dyskinesia, which can then be used as outcome variables to assess the effects of interventions in advanced PD.

In recent years, clinical studies have repeatedly used the PD home diary as primary outcome measure to assess effects of novel treatments on motor fluctuations in advanced PD, such as opicapone^6^, rasagiline^7,8^, safinamide^9^, subcutaneous apomorphine^10^ or levodopa-carbidopa intestinal gel^11^. Despite its widespread use, the diary has however not been extensively validated against direct clinical observation, which despite the rise of wearable sensor technology ultimately remains the gold standard for objectifying motor fluctuations. We therefore performed a study, which was designed to compare patient diary ratings of motor states to direct clinical assessments of an experienced observer. Based on our daily clinical experience, we hypothesized that patient diaries would only suboptimally reflect actual motor states in patients with advanced PD.

## Results

### Demographic and clinical data

Between October 2017 and July 2019, we screened 55 patients for eligibility of whom 51 were successfully included into the study. Four participants were excluded since they reached Montreal Cognitive Assessment (MoCA) scores below 21 points at screening (*n* = 2) or were not able to sufficiently adhere to diary assessments (*n* = 2). Demographic and clinical characteristics of the cohort are displayed in Table 1. In brief, complete datasets were available from 26 male (51%) and 25 female (49%) PD patients with a median age of 65 years. Participants had been diagnosed with PD about 10 years prior to the study and were suffering from motor fluctuations since 61 months. During structured interviews, patients reported a wide range of motor fluctuations, in particular wearing-off (94.1%), nocturnal off (82.4%) and peak-dose dyskinesia (76.5%). Clinical scores and antiparkinsonian medication were representative for a patient population suffering from advanced PD (Table 1).

**Table 1:** Demographics and clinical characteristics of the study cohort (*n* = 51).

Male/Female	26 (51%)/25 (49%)
Age, median (IQR) in years	65 (57–72)
Disease duration, median (IQR) in years	10 (8–15)
Symptom duration, median (IQR) in years	12 (9–17)
Duration of fluctuations, median (IQR) in months	61 (37–109)
Hypokinetic fluctuations	78 (42–130)
Hyperkinetic fluctuations	38 (25–56)
*Clinical phenotype*	
Tremor dominant (TD)	8 (16%)
Axial dominant (AxD)	0 (0%)
Appendicular dominant (ApD)	3 (6%)
Rigor dominant (RD)	1 (2%)
Postural instability and gait difficulty (PIGD)	39 (77%)
*Laterality of symptoms*	
Right	25 (49%)
Left	22 (43%)
No laterality	4 (8%)
*Reported motor complications during structured interview*	
Nighttime off	42 (82%)
Wearing-off	48 (94%)
Delayed on	38 (75%)
On–off phenomenon	31 (61%)
Peak-dose dyskinesia	39 (77%)
Biphasic dyskinesia	10 (20%)
Off-dose dystonia	27 (53%)
*Clinical scales*	
MDS-UPDRS total score in on state, median (IQR)	64 (52–82)
Part I (Non-motor aspects of experiences of daily living)	13 (8–16)
Part II (Motor aspects of experiences of daily living)	14 (10–20)
Part III (Motor examination)	28 (21–39)
Part IV (Motor complications)	9 (7–10)
Hoehn & Yahr stage, median (IQR)	3 (2–3)
Montreal cognitive assessment, median (IQR)	27 (25–28)
Beck Depression Inventory Second Edition, median (IQR)	9 (4–15)
*Anti-Parkinsonian medication*	
Levodopa	51 (100%)
Catechol-O-methyl transferase inhibitors	45 (88%)
Dopamine agonists	33 (65%)
Monoamine oxidase B inhibitors	35 (69%)
Amantadine	25 (49%)
Levodopa dose, mean (SD) in mg	582.2 (219.0)
Levodopa equivalent dose, mean (SD) in mg	1371.4 (446.5)

Values are provided as number (percentages), median (interquartile range, IQR), or mean (standard deviation, SD). MDS-UPDRS: Movement Disorder Society-revised version of the Unified Parkinson’s Disease Rating Scale. Levodopa equivalent doses were calculated according to Tomlinson et al.^28^.

### PD home diary data

In total, we examined 1997, 30-min intervals with simultaneous ratings of motor states on the patient and the observer diary. Agreement between patient and observer ratings was found in 59.8% of diary entries with a Cohen’s kappa of 0.387. Multivariate linear regression analysis including age, gender, disease duration, duration of motor fluctuations, Beck Depression Inventory II (BDI-II) score and MoCA score as independent variables for the prediction of diary agreement revealed a significant association of BDI-II scores (*P* < 0.002) and MoCA scores (*P* < 0.002) with individual diary agreement rates (Supplementary Fig. 1).

**Fig. 1:**
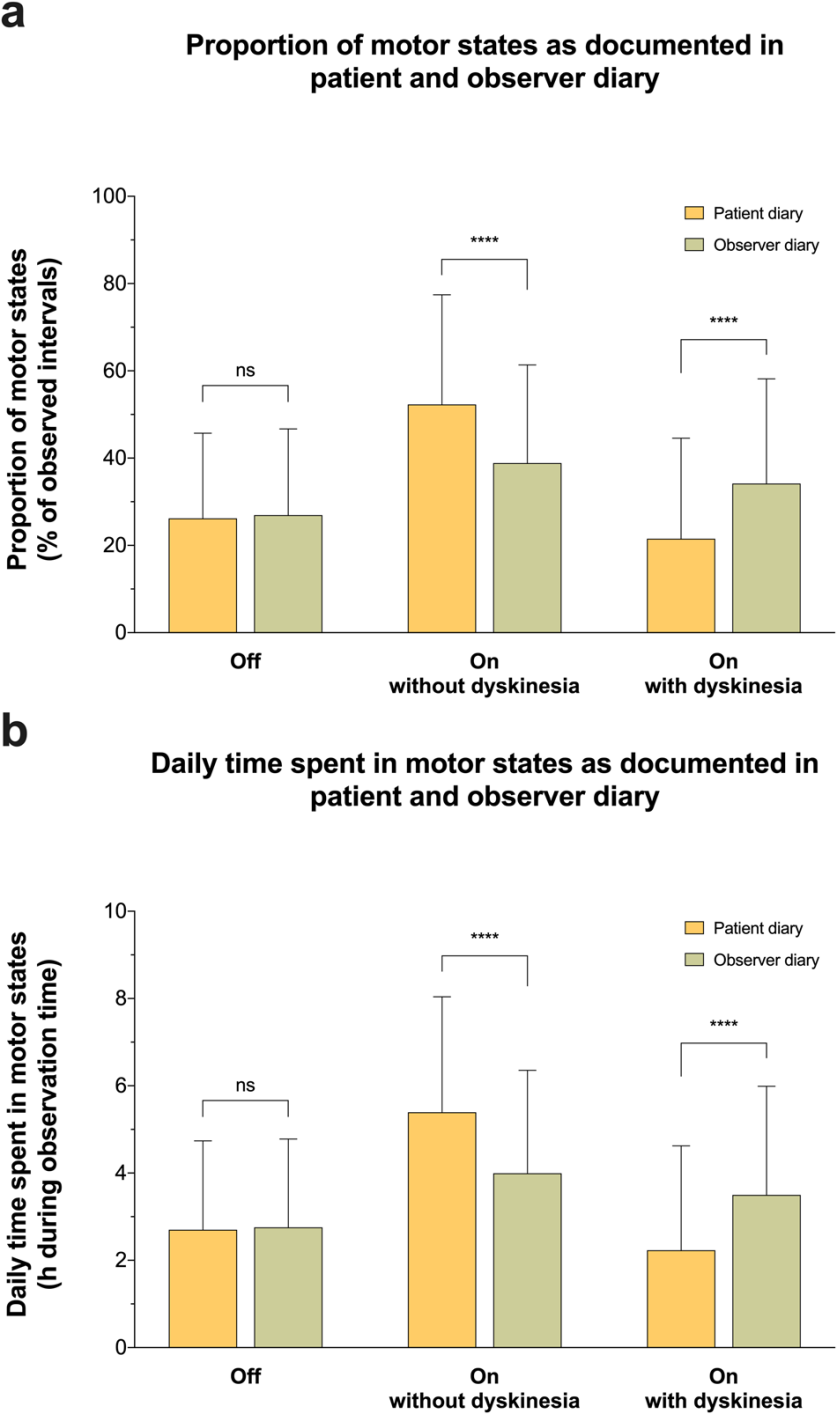
Proportion of motor states assessed by patient and observer diaries. **a** The mean proportions of Off, On without dyskinesia and On with dyskinesia based on 1997 simultaneous, half-hourly performed diary ratings from 51 patients with Parkinson’s disease (yellow colour) and an independent clinical observer (green colour). **b** Illustrates the daily time spent in the respective motor states during the observation period as documented on the patient (yellow colour) and on the observer diary (green colour). Values are provided as means + standard deviation. ****P < 0.0001 from Wilcoxon matched-pairs signed rank tests. ns not significant.

In comparison to the observer, patients documented significantly more On without dyskinesia (52.3% vs. 38.9%, *P* < 0.001) and less On with dyskinesia (21.5% vs. 34.2%, *P* < 0.001), whereas proportions for Off intervals were not different between the patient and the observer diary (26.2% vs. 27.0%, *P* = 0.97; Fig. 1a). Corresponding differences were found when we calculated the absolute times spent in individual motor states with respect to diary entries during the observation period (Fig. 1b). Patients reported more On time without dyskinesia (5.4 h vs. 4.0 h, *P* < 0.001) and less On time with dyskinesia (2.2 h vs. 3.5 h, *P* < 0.001) than the observer, whereas Off time was similar between patient and observer diaries (2.7 h vs. 2.8 h, *P* = 0.97). Ancillary analyses of the diaries incorporating the original categories “On with troublesome dyskinesia” and “On with non-troublesome dyskinesia” did not reveal different results (Supplementary Fig. 2).

**Fig. 2:**
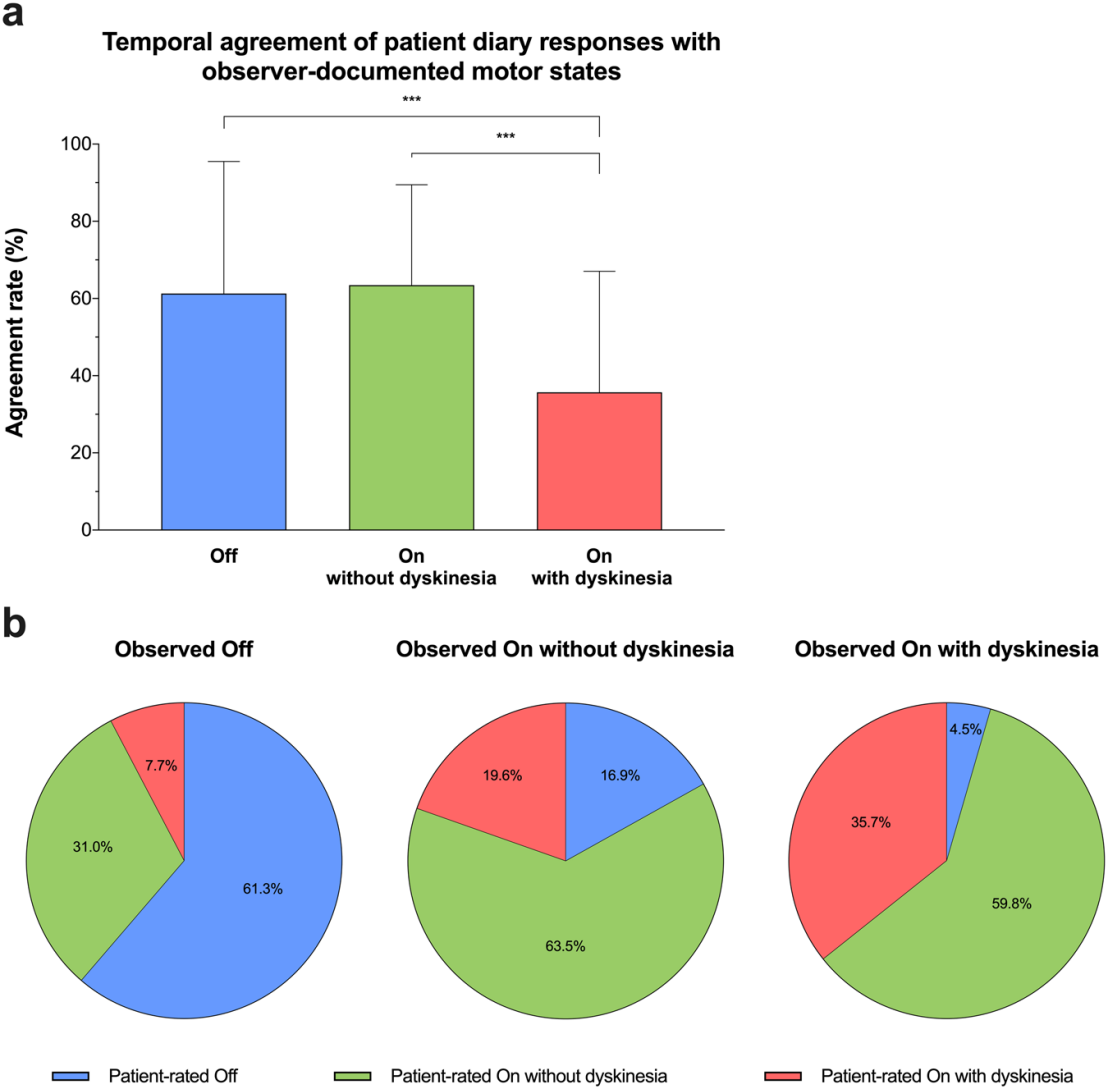
Temporal agreement of patient diary responses with observer-documented motor states and preferred patient choices in the respective motor states. **a** The mean agreement rate for Off (blue colour), On without dyskinesia (green colour) and On with dyskinesia (red colour) based on 1997 simultaneous half-hourly diary ratings of 51 patients with Parkinson’s disease and an independent clinical observer serving as reference for the comparison. **b** Illustrates preferred choices on the patient diary in the respective motor states. Values provided as means + standard deviation (**a**) and proportions in % (**b**). ***P < 0.001 from Kruskal–Wallis test with Dunn–Bonferroni post-hoc tests, corrected for multiple comparisons.

Taking the observer diary as gold standard, we next estimated temporal agreement rates for individual motor states. Patients recognized 61.3% of observed Off states, 63.5% of On states without dyskinesia, but only 35.7% of On states with dyskinesia simultaneous to the observer (Fig. 2a). Temporal agreement rates for Off and for On without dyskinesia were significantly higher than for On with dyskinesia (both *P* < 0.001). Patients considered themselves to be On in 38.7% of the intervals with observed Off (Fig. 2b). Even more strikingly, patients chose On without dyskinesia in 59.8% of those intervals in which the observer had actually noted dyskinesia. Additionally calculated intraclass correlation coefficients (ICC) for the comparison of patient and observer diary responses revealed only modest ICCs ranging from 0.64 for daily Off-time to 0.50 for daily On-time with dyskinesia, suggesting only moderate reliability of the PD home diary in the assessment of motor states (Supplementary Table 1). Ancillary analyses investigating the timing of Off ratings moreover suggested that patients partially depicted Off states prior to the clinical observer and before worsening of motor performance on the Timed Up and Go test actually occurred (Supplementary Fig. 3).

### 7-meter Timed Up and Go Test (7m-TUGT) data

**Fig. 3:**
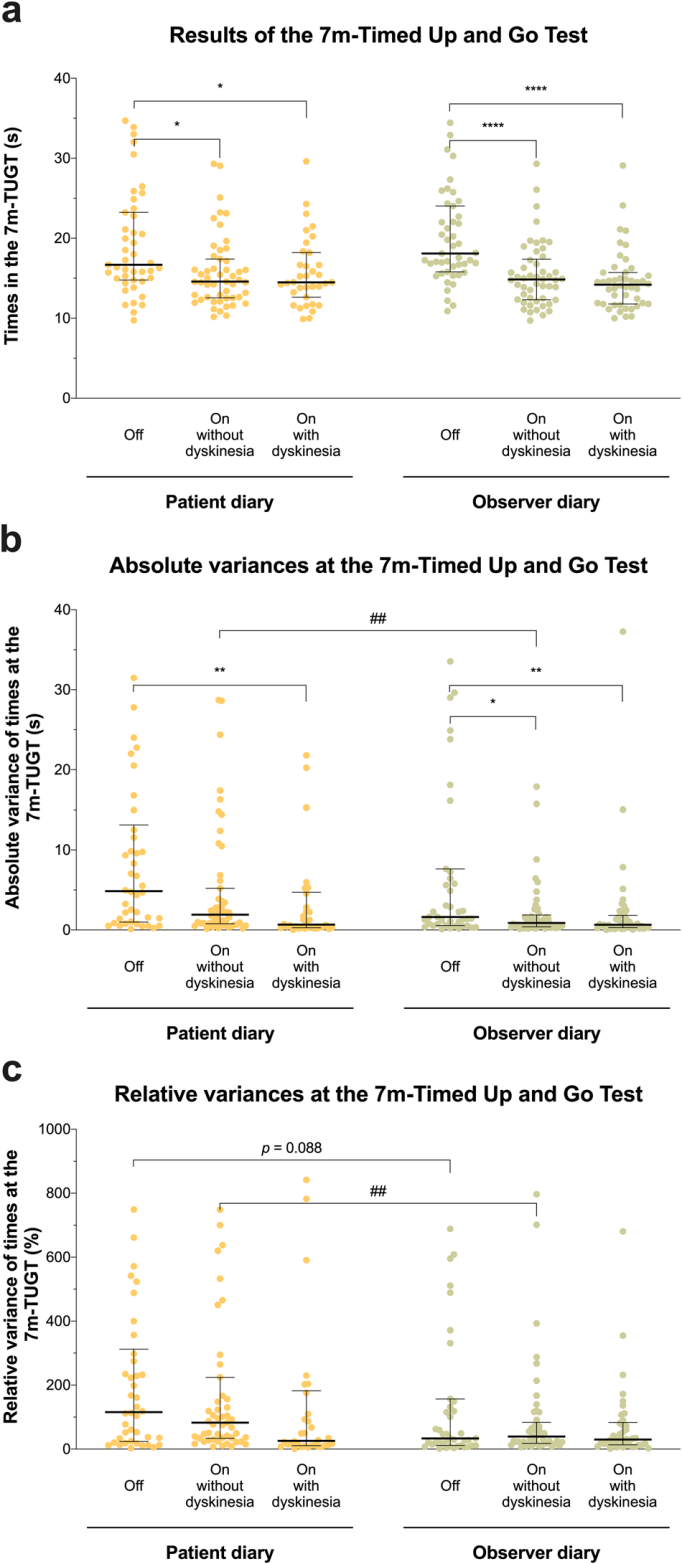
Results from 7-meter Timed Up and Go Tests with respect to diary-documented motor states. **a** The results of 1917 half-hourly performed 7-meter Timed Up and Go Tests (TUGT) in 51 patients with Parkinson’s disease with respect to Off, On without dyskinesia and On with dyskinesia as documented in the patient diary (left hand side, yellow colour) and observer diary (right-hand side, green colour). **b** Illustrates absolute variances during the 7-m TUGT in respective motor states. **c** Displays relatives variances after individual normalization of measured 7-m TUGT times to the mean time achieved by a patient in the respective motor state. Single dots indicate mean values for individual patients, black bars and whiskers represent medians ± interquartile ranges for the entire cohort. *P < 0.05, **P < 0.01 and ****P < 0.0001 from Kruskal–Wallis tests with Dunn–Bonferroni post-hoc tests, corrected for multiple comparisons. ^##^*P* < 0.01 from Mann–Whitney test. 1 (**a**), 7 (**b**) and 11 (**c**) data points are not shown to allow for proper scaling of the graphs.

We performed 1917 half-hourly 7m-TUGT to externally validate the ratings in the patient and the observer diary against a standardized clinical test. After grouping 7m-TUGT results with respect to corresponding entries in the motor diaries, we found significantly higher 7m-TUGT times for Off intervals compared to On without dyskinesia and On with dyskinesia for both patient (*P* < 0.05) and observer diary (*P* < 0.0001; Fig. 3a). Comparison of absolute variances of 7m-TUGT times in individual motor states showed significantly lower variances for On states without dyskinesia that had been rated by the clinical observer than for patient-rated On without dyskinesia (*P* < 0.001; Fig. 3b), arguing for higher consistency in the ratings of the observer. Similarly, we found lower variances of relative 7m-TUGT times for On with dyskinesia with the observer diary (*P* < 0.001) and a comparable trend for Off intervals (*P* = 0.088; Fig. 3c).

### Patient Global Impression of Severity (PGI-S) and Clinical Global Impression of Severity (CGI-S) data

**Fig. 4:**
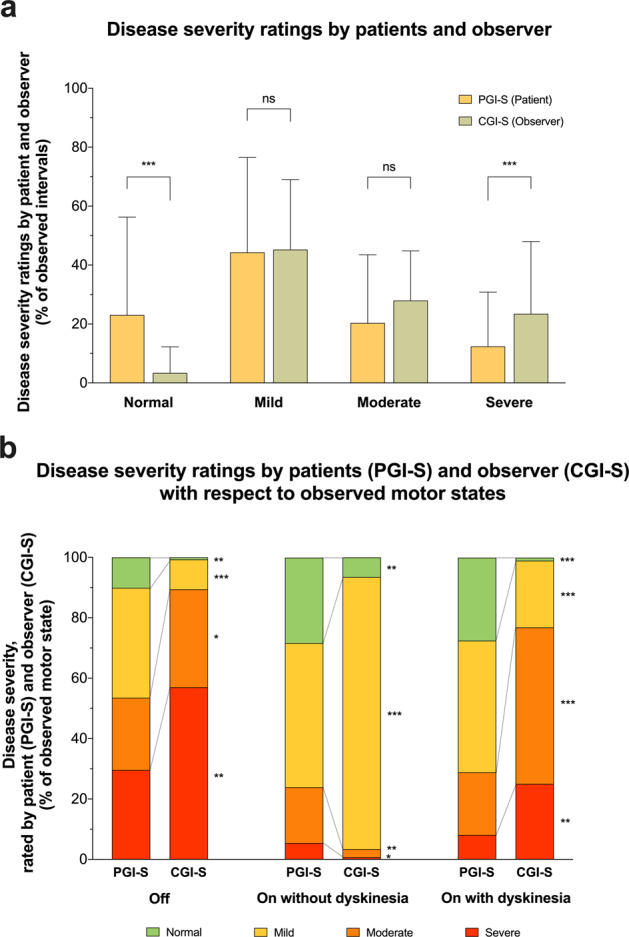
Perception of disease severity by patients and observer on the Patient Global Impression of Severity (PGI-S) and Clinical Global Impression of Severity (CGI-S). **a** The mean proportions of 1947 simultaneous half-hourly performed disease severity ratings from 51 patients with Parkinson’s disease (yellow colour) and an independent clinical observer (green colour) using the Patient Global Impression of Severity (PGI-S) and Clinical Global Impression of Severity (CGI-S), respectively. **b** Illustrates the distribution of PGI-S and CGI-S ratings with respect to correspondingly observed motor states using the levels “Normal” (green colour), “Mild” (yellow colour), “Moderate” (orange colour) and “Severe” (red colour). All PGI-S and CGI-S values were originally each assessed with 7 severity grades, but then condensed to four levels to enhance clarity of this figure (please refer to the “Methods” section for more details). Values are means + standard deviations (**a**) and percentages **b**. *P < 0.05, **P < 0.01, and ***P < 0.001 from Wilcoxon matched-pairs signed rank. ns not significant.

The half-hourly motor ratings in our study were complimented by 1947 simultaneous ratings of PGI-S and CGI-S to explore differences in the perception of disease severity between patients and observer across different motor states. Agreement between the PGI-S and CGI-S was noted in 35.1% of diary entries, yielding a Cohen’s kappa of only 0.089. In comparison to the observer, patients more often judged themselves to be normal (23.0% vs. 3.4%, *P* < 0.001) and less often found themselves severely affected by the disease (12.4% vs. 23.5%, *P* < 0.001; Fig. 4a). Similar differences were found when we analysed the PGI-S and CGI-S data in the originally collected format with 7 severity grades on both scales (Supplementary Fig. 4).

Moreover, there were striking differences in disease perception between patients and observer across all motor states when we grouped PGI-S and CGI-S ratings with respect to simultaneously documented motor states (Fig. 4b). Patients found themselves to be severely affected in only 29.6% of Off intervals and 8.1% of intervals with dyskinesia, whereas the observer selected “severe” on the CGI in 57.0% and 25.0% in the respective motor states (both *P* < 0.01). Conversely, patients rated themselves to be normal on the PGI-S in 10.1% of documented Off periods and in 27.5% of intervals with observed dyskinesia, which was contrasted by 0.7% and 1.1% on the CGI-S ratings by the observer (*P* < 0.01 and *P* < 0.001, respectively; Fig. 4b).

## Discussion

We report a prospective, observational cohort study in 51 patients with advanced PD investigating the validity of the PD home diary for the assessment of motor fluctuations. By comparing patient responses on the PD home diary to simultaneous assessments of a clinically experienced observer, we found good agreement with respect to total Off time but significant differences in the proportions of On without dyskinesia and On with dyskinesia. Moreover, temporal agreement between patient dairy responses and the observer was insufficient, particularly for On with dyskinesia. These discrepancies in motor ratings were accompanied by striking differences in the perception of disease severity in respective motor states between patients and observer.

The current version of the PD home diary by Hauser et al. has originally been proposed after a study based on valid diaries from 17 PD patients with motor fluctuations had shown that the categorization into “Off”, “On without dyskinesia”, “On with non-troublesome dyskinesia” and “On with troublesome dyskinesia” allowed for sufficient separation between “good time” and “bad time” on a self-completed reference diary^4^. The PD home diary was then further evaluated in a prospective, international study to investigate its reliability and perform sample size calculations for clinical trials^3^ and later complemented by an instructional video and pictograms illustrating the functional states^5^, which we both adopted for our study. Validation in previous studies was however limited to comparisons with self-rated disease perception in reference diaries^4^ or visual analogue scales^3^, whereas the accuracy of patient responses was not externally validated against direct clinical observation as in our study. Despite the use of an instructional video, an adopted diary with pictograms and one run-in day for diary training, we only found good agreement for average daily Off-time, whereas proportions for On without dyskinesia and for On with dyskinesia were significantly different between patient and observer diaries. This discordance is in keeping with studies showing greater awareness and psychological suffering in the Off state than in the On state^12^ and reduced awareness for dyskinesias in PD^12–15^, which has been attributed to dysregulation of frontal-subcortical loops due to dopaminergic overstimulation of mesocorticolimbic pathways^12,15,16^ and anosognosia due to right hemisphere dysfunction^17,18^. In addition, one might speculate that Off states are more reliably recognized by patients due to accompanying non-motor symptoms, which have been shown to be more frequent and severe during Off states^19,20^. Our ancillary analyses regarding the synchrony of Off ratings revealed that patients partly documented Off prior to the clinical observer and before objective worsening of motor performance on the 7m-TUGT occurred, suggesting that prodromal non-motor symptoms may have contributed to the poor temporal agreement between patients and observer ratings of motor states. Taken together, our data suggest that it is difficult for patients to dissect their current motor status from prodromal and/or simultaneous non-motor symptoms while using the diary. Unlike originally intended, patient ratings on the PD home diary are thus not providing a sole reflection of motor function, but most likely are also influenced by the presence of non-motor symptoms and/or subjective well-being of a patient.

Furthermore, our study identified a profoundly altered disease perception in advanced PD patients. For example, patients perceived themselves to be completely normal in 10.1% of Off intervals and 27.5% of intervals with observed dyskinesia. Interestingly, similar misperceptions during periods with Off or dyskinesia have already been reported in the original evaluation study by Hauser and colleagues and then attributed to patient errors, changes of the clinical status within the half-hour intervals or differential influences of non-motor symptoms on disease perception^4^. Since we found discrepancies in the judgement of disease severity across all motor states including On states without dyskinesia, it seems likely that there is a generally altered perception of normality in PD patients as the disease progresses, which ultimately hinders patients to objectively rate their motor status and argues against the use of self-rated disease perception for external validation of motor diaries as applied in previous studies^3,4^. While cross-sectional studies have reported reduced self-awareness of motor deficits^12–15,17,18^ and cognitive dysfunction^21,22^, it still remains to be elucidated how individual perception of symptoms and normality changes in the longitudinal course of the disease and how this effects self-reported outcomes in clinical trials.

The results of our study have several practical implications. While absolute Off-time seems to be adequately reflected by the PD home diary, the misrepresentation of On-time should be taken into account when interpreting double-blind randomized clinical trials in advanced PD, which in the past reported On-time without troublesome dyskinesia as primary^9^, secondary^6,10,11^ or exploratory^7,8^ outcome measure to demonstrate the efficacy of novel drugs on motor fluctuations. Moreover, our study clearly underscores the metric limitations of paper diaries and reiterates the need for development of a patient-friendly and intuitive electronic diary, which should be capable to simultaneously assess both motor and non-motor fluctuations and use action-dependent and action-independent end points as recently suggested by the MDS Technology Task Force and the MDS Rating Scales Programme Electronic Development Ad-Hoc Committee^23^. Ultimately, the use of technology-based measurement tools, such as wearable devices, bears the potential to eliminate the subjectivity that is associated with patient and observer ratings and could complement existing clinical measures by providing objective, quantifiable scores of motor function^24^.

We took several precautions to ensure a high validity of our study results. First, we trained all patients how to depict motor fluctuations prior to the diary recordings. We also used an instructional video to enhance understanding of different motor states and utilized an adopted version of the PD home diary with pictograms, which has shown to be preferred by patients in comparison to the original version^5^. Secondly, we used a single-rater approach with one specifically trained clinical observer to exclude potential bias by inter-rater variability on observer ratings. Thirdly, we validated all patient and observer diary responses against the results of a simultaneously adjudicated 7m-TUGT. External validation of both diaries against this standardized clinical test showed better separation of 7m-TUGT results between Off and On states and a lower variability during On without dyskinesia with the observer diary argueing for a high consistency and reliability of observer ratings, even though they might not always have reflected those of an established movement disorders specialist. Our study also has some limitations. For example, one could argue that the ratings of the observer might not have been entirely based on clinical judgement of symptoms as intended by the protocol, but have been facilitated by simultaneously recording 7m-TUGT times. This would however have required a priori knowledge of patient-specific 7m-TUGT times in individual motor states, which was not applicable to the observer in our study, since he had not examined the patients before ratings started. Secondly, the use of an in-patient cohort from a two centres specialized in movement disorders could limit generalizability of our results to a greater population. Nonetheless, strikingly similar observations have been made by a parallel study with outpatients in Sweden with an adapted version of our protocol, which also showed discrepancies between patient diaries and observed motor states^25^. Thirdly, we intentionally excluded patients who screened positive for dementia to ensure proper understanding of motor states and adherence to the half-hourly ratings. Hence, our results may not be representative and are likely to be even worse for PD patients with profound cognitive dysfunction.

Together, our study suggests that the PD home diary insufficiently reflects actual motor states and that temporal agreement of patient diary responses with direct clinical observation is unsatisfactory, particularly for dyskinesia. So far, the use of the PD home diary in clinical trials nonetheless remains standard to investigate effects of interventions on motor fluctuations in advanced PD, especially since better assessment tools for motor function are yet to be established^23^. Future studies need to show whether electronic diaries in conjunction with wearable sensor technology will be capable to provide more objective and at the same time clinically meaningful outcomes in PD patients with motor fluctuations.

## Methods

### Study protocol approvals and patient consents

This prospective, observational cohort study (VALIDATE-PD) was conducted at two hospital centres in Germany (University Medicine Rostock, Movement Disorder Clinic Beelitz-Heilstätten) between October 2017 and July 2019. The study was approved by the institutional review boards of both participating centres (ethic committee registry numbers A 2017-0115 for Rostock and AS 84(bB)/2018 for Beelitz-Heilstätten). All participants provided written informed consent before study participation.

### Participants

Participants were eligible for the study if they were over 30 years old, had been diagnosed with PD according to the United Kingdom PD Society Brain Bank criteria, suffered from motor fluctuations observed by the treating physician and/or documented on part 4 of the Movement Disorder Society-revised Unified Parkinson’s Disease Rating Scale (MDS-UPDRS) and were able to provide written informed consent.

Exclusion criteria comprised the existence of any clinical signs for secondary or atypical parkinsonian syndromes, inability to complete questionnaires and/or patient diaries, lack of cooperation during the study procedures, presence of dementia (defined as scores on the Montreal Cognitive assessment (MoCA) below 21)^26^ and/or relevant psychotic symptoms, ongoing treatment with advanced/invasive therapies (deep brain stimulation, subcutaneous apomorphine and levodopa-carbidopa intestinal gel) as well as the presence of miscellaneous diseases impairing the ability for consenting, participation and judgement in the patient.

### Assessments

The study commenced with a detailed screening, which included cognitive screening with the MoCA, clinical evaluation using the MDS-UPDRS and assessment of non-motor symptoms using the Non-Motor Symptom Scale (NMSS) and the King’s Parkinson’s Disease Pain Scale (KPDPS). Furthermore, all participants were asked to fill-out the 19-item Wearing-off Questionnaire (WOQ-19), Non-Motor Symptoms Questionnaire (NMSQuest), King’s PD Pain Quest, Beck Depression Inventory version 2 (BDI-II) and 39-item Parkinson’s disease Questionnaire (PDQ-39) for ancillary analyses.

After inclusion into the study, all participants received detailed instructions on the PD home diary and watched a training video explaining all functional states with particular focus on the difference between tremor and dyskinesia^5^. Participants were then asked to indicate their predominant status during half-hour time periods for three consecutive days using the categories Asleep, Off, On without dyskinesia, On with non-troublesome dyskinesia, and On with troublesome dyskinesia.

The initial day (day 0) was used for diary training and to ensure sufficient adherence to the motor diary. Participants were visited by their treating neurologists, who checked diaries for inconsistencies and addressed potential questions.

On the following two days (day 1 and 2), participants were observed by an experienced physiotherapist (A.B.), who had been trained to identify motor complications in advanced PD patients in the participating hospitals and acquired MDS certification as qualified UPDRS rater prior to the start of the study. The observer acted as single rater in our study and independently evaluated motor states half-hourly throughout daytime (min. 8 AM through 6 PM) based on his clinical observations during a 7-meter version of the Timed Up and Go test (7m-TUGT)^27^, taking into account global bradykinesia, tremor, dyskinesia and gait function. All 7m-TUGT times were recorded but not considered for the observer ratings. The observer was also instructed to dismiss any attempts from patients to get assistance with their own ratings.

In addition to the motor diaries, patients and observer rated disease severity in individual motor states half-hourly using 7-point versions of Patient Global Impression of Severity scale (PGI-S) and Clinical Global Impression of Severity scale (CGI-S), respectively, ranging from “normal” to “extremely ill”. Moreover, patients were filling out specifically developed non-motor diaries, which are not subject of this paper.

### Statistics

Statistical analysis was performed using IBM SPSS Statistics software version 27 (IBM Corporation, New York, USA). Values are provided as means (standard deviation, SD) or median (interquartile range, IQR), as appropriate. Pairwise exclusion was used for missing values. As most of the data was not normally distributed, we chose non-parametric tests for statistical comparisons. Adjustment for multiple comparisons was performed as indicated below. *P* values < 0.05 were considered to be statistically significant.

#### Motor diary data

Statistical analysis of the diary data was confined to all 30 min periods, for which simultaneous ratings from patients and observer had been recorded. While diary data for both participants and the observer was initially collected using the categories originally defined by Hauser et al.^4^, we eventually combined the categories “On with non-troublesome dyskinesia” and “On with troublesome dyskinesia” into the category “On with dyskinesia” for analysis, since the distinction between non-troublesome and troublesome dyskinesia could only be made by patients. Overall diary agreement for the choice between the three categories Off, On without dyskinesia and On with dyskinesia between participants and observer was calculated as percentage and Cohen’s kappa. To further investigate the influence of clinical variables on diary agreement, we additionally performed multivariate linear regression for individual agreement rates including age, gender, disease duration, duration of fluctuations, BDI-II score and MoCA score as independent variables, using a step-wise selection with *P* < 0.05 for adding and *P* < 0.10 for removing variables.

To examine the patients’ ability to identify individual motor states, we assumed the ratings of the independent observer as gold standard and calculated temporal agreement rates for Off, On without dyskinesia and On with dyskinesia, which referred to the proportion of 30-min intervals in which the choice in the patient diary was in agreement with an observed motor state. Agreement rates between motor states were compared with the Kruskal–Wallis test using Bonferroni correction for multiple comparisons. Pie diagrams were plotted to illustrate the patients diary choice in observed motor states.

In addition, we analysed the temporal connection between Off periods rated by the objective observer and Off intervals as indicated by patients on the PD home diary. For this ancillary analysis, we selected Off periods of at least 30 min duration, which were following a motor On period of at least 90 min to exclude sequential on-off fluctuations of short duration (“yo-yoing”). These selected Off periods were then synchronized by summation of all Off intervals as rated by the objective observer using the first 30 min of the motor Off period as the trigger event (start of Off period). The Off state ratings from PD diaries were then cross-classified by putting them into 2 × 2 contingency tables for each 30-min motor Off state interval. All diary sets were included for analysis. Individual time periods were excluded from analysis if there was no response or more than one response on either diary, or if the patient indicated they were asleep.

#### TUGT data

We used the results of half-hourly performed 7m-TUGT to validate patients and observer ratings against an objective and unobtrusive clinical test. For this, we first grouped all recorded TUGT times with respect to the corresponding entry in the patient and observer diary and then calculated the mean 7m-TUGT times for Off, On without dyskinesia and On with dyskinesia for each participant based on the patient and the observer diary ratings, respectively. For the entire cohort, results were summarized as median and interquartile ranges to minimize bias by outliers. In order to compare the consistency of measured TUGT times with respect to patients and observer ratings, we additionally estimated variances for absolute and relative 7m-TUGT times for each motor state. For the latter, we first calculated a patient-specific mean 7m-TUGT time in a given motor state, computed relative values for all TUGT times with respect to that mean value and then examined the variance over all relative values in this motor state for each individual patient. Comparisons of 7m-TUGT times and their variances within the three motor states were performed with the Kruskal–Wallis test using Bonferroni correction for multiple comparisons, whereas 7m-TUGT times in corresponding motor states between patient and observer diaries were compared with the Mann–Whitney *U*-test.

#### PGI-S and CGI-S data

Similar to the data from the motor diaries, statistical analysis of the PGI-S and CGI-S was performed for all 30 min periods, for which simultaneous ratings from patients and observer had been collected. For simplicity, we condensed the original 7 grades of disease severity on both scales to four categories: “normal” (containing the original grade ‘normal’), “mild” (comprising the original grades ‘borderline ill’ and ‘mildly ill’), “moderate” (containing the original grade ‘moderately ill’) and “severe“ (comprising the original grades ‘profoundly ill, ‘severily ill’ and extremely ill’). To compare perceived disease severity by patients and observer in different motor states, we grouped all PGI-S and CGI-S responses with respect to the motor state simultaneously documented by the observer. Comparison between PGI-S and CGI-S proportions in individual motor states was then made with the Mann–Whitney *U*-test.

## Supplementary information

Supplementary Materials

## Data Availability

All data that support the findings of this study are available upon reasonable request from the corresponding author.
