# Supplementary material for: Validation of the PD home diary for assessment of motor fluctuations in advanced Parkinson’s disease
Source: NPJ Parkinsons Dis. 2022 Jun 2;8:69. doi: 10.1038/s41531-022-00331-w (PMC9163037; doi:10.1038/s41531-022-00331-w)
Supplement: Supplementary file 1 — Supplementary Materials [file 41531_2022_331_MOESM1_ESM.pdf]

Supplementary Information for the manuscript:

Validation of the PD Home Diary for Assessment of Motor Fluctuations in Advanced Parkinson's Disease

Matthias Löhle, Alexander Bremer, Florin Gandor, Jonathan Timpka, Per Odin, Georg Ebersbach, and Alexander Storch

Supplementary Table 1 | Reliability of the PD home diary according to intraclass correlation coefficient (ICC) calculation[#]

**Patient vs. observer diary**	**ICC (95%CI)**	**F-Test with true value 0**			
		**Value**	***df1***	***df2***	**P value**
**Proportion of motor states**					
Off	0.65 (0.46-0.79)	4.66	50	50	<0.001
On without dyskinesia	0.54 (0.21-0.74)	4.19	50	50	<0.001
On with dyskinesia	0.52 (0.21-0.72)	3.85	50	50	<0.001
**Daily time spent in motor states**					
Off	0.64 (0.45-0.78)	4.51	50	50	<0.001
On without dyskinesia	0.52 (0.19-0.72)	3.91	50	50	<0.001
On with dyskinesia	0.50 (0.21-0.70)	3.60	50	50	<0.001

[#]ICC estimates and 95%CIs were calculated based on single-rating, absolute-agreement, 2-way mixed-effects models with two raters (patient diary and observer diary) across 51 participants.

a

**Independent variables**	**B**	**SE (B)**	**Beta**	**t**	***P***
Constant	-8.783	26.127		-0.336	0.738
Age (years)	0.351	0.211	0.256	1.664	0.103
Gender (male/female)	-6.438	3.410	-0.260	-1.888	0.066
Disease duration (months)	-0.027	0.031	-0.143	-0.875	0.386
Duration of motor fluctuations (months)	0.002	0.031	0.012	0.078	0.938
Beck Depression Inventory II (points)	0.911	0.272	0.455	3.352	0.002
Montreal Cognitive Assessment (points)	2.013	0.739	0.375	2.726	0.009
	**R^2**	**Adj.**	**SE**	**F**	***P***
**Model**	0.289	0.192	11.262	2.974	0.016

b

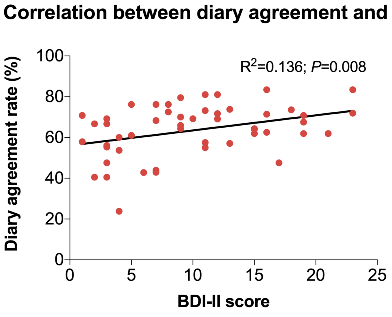

c

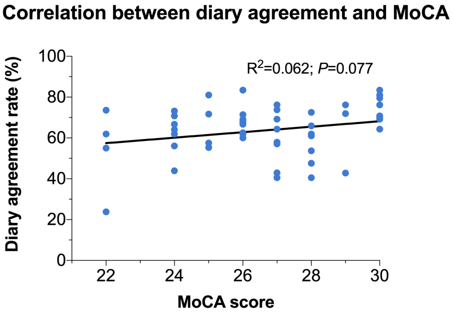

Supplementary Figure 1 | Multiple linear regression analysis for demographic and clinical variables predicting the individual mean agreement of patient and observer diary ratings of motor fluctuations. Subfigure a provides the results of a multiple linear regression using the independent variables age, gender, disease duration, duration of motor fluctuations, Beck Depression Inventory Version 2 (BDI-II) score and Montreal Cognitive Assessment (MoCA) score for prediction of the individual mean agreement rate of patient and observer diary ratings on motor fluctuations. Subfigure b illustrates the correlation between individual BDI-II scores and the mean diary agreement rate (red dots). Subfigure c depicts the correlation between individual MoCA scores and the mean diary agreement rate (blue dots). B: Unstandardized regression coefficient. SE (B): Standard error of the unstandardized regression coefficient. Beta: Standardized regression coefficient. T: t value. R2: R-square, coefficient of multiple determination. Adj. R2: Adjusted R-square. SE: Standard error of the estimate. F: F value. *P*: P value.

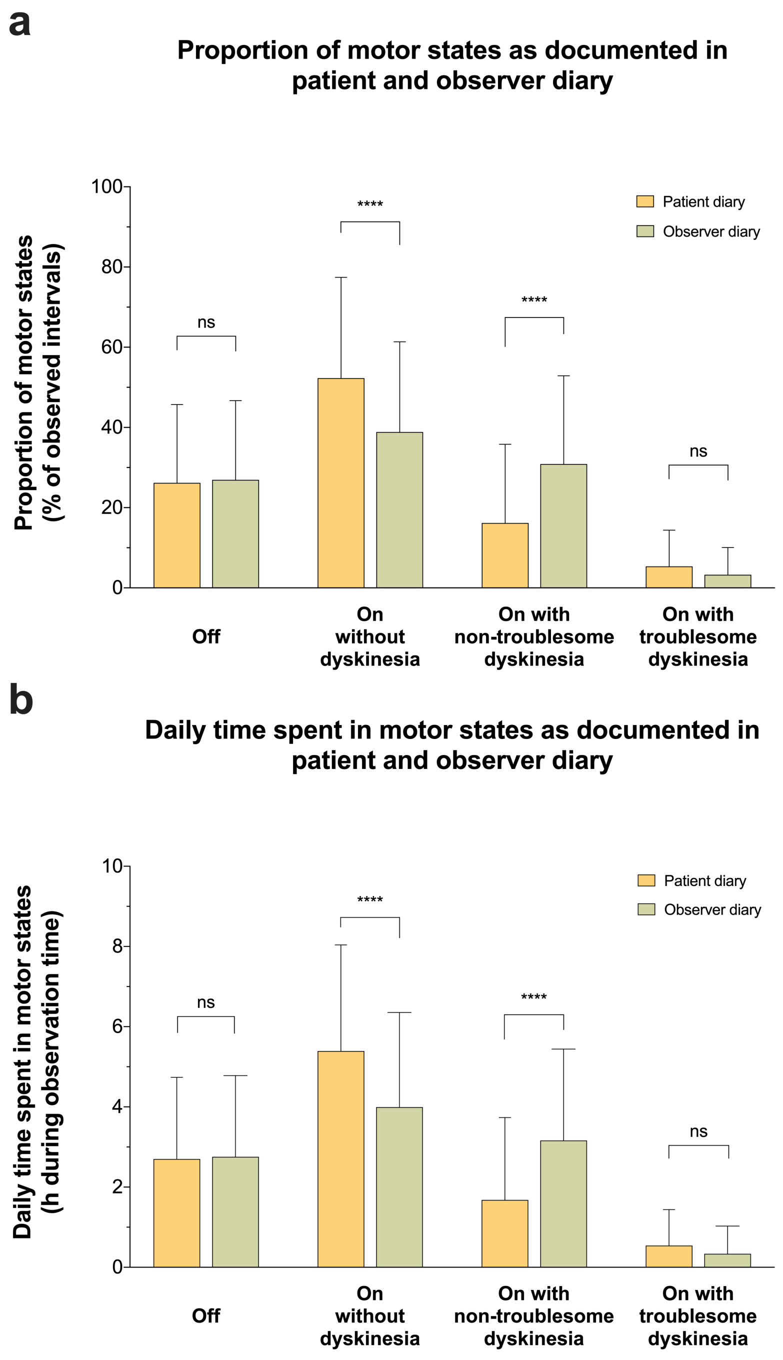

Supplementary Figure 2 | Proportion of motor states assessed by patient and observer diaries. Subfigure a shows the mean proportions of Off, On without dyskinesia, On with non-troublesome dyskinesia and On with troublesome dyskinesia based on 1997 simultaneous, half-hourly performed diary ratings from 51 patients with Parkinson's disease (yellow colour) and an independent clinical observer (green colour). Subfigure b illustrates the daily time spent in respective motor states during the observation period as documented in the patient (yellow colour) and the observer diary (green colour). Values are provided as means + standard deviation. ****$P<0.0001$ from Wilcoxon matched-pairs signed rank tests. ns: not significant.

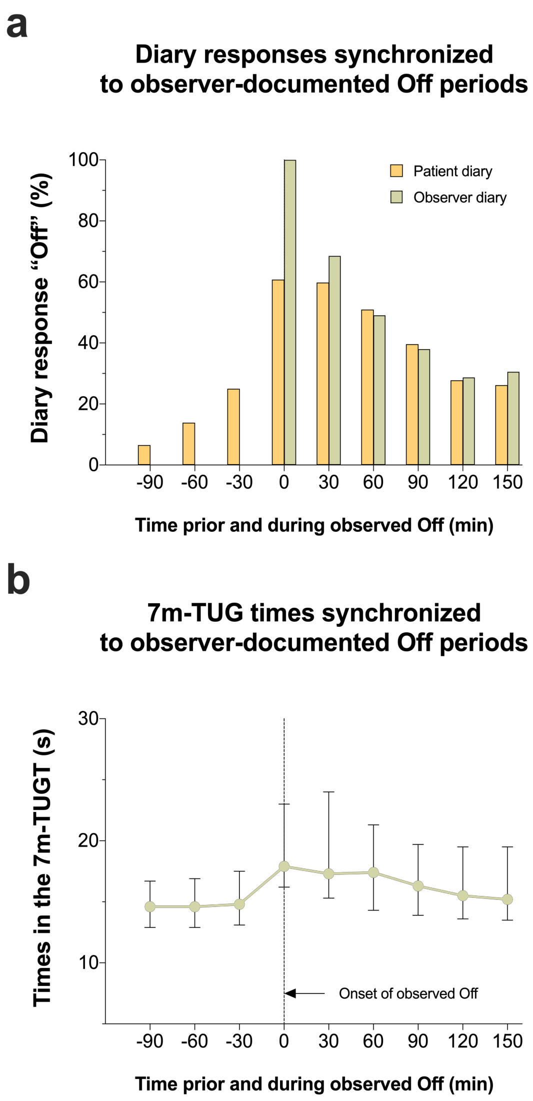

Supplementary Figure 3 | Diary responses and results from 7-meter Timed Up and Go tests synchronized to the onset of observed Off periods. Subfigure a shows the proportions of Off responses on simultaneous diary ratings from 51 patients with Parkinson's disease (yellow colour) and an independent clinical observer (green colour) synchronized to the onset of 108 observed Off periods, defined as a minimum of 30 min Off preceded by at least 90 min with On time as judged by the clinical observer. Subfigure b displays times from 7-meter Timed Up and Go tests synchronized to 108 observed Offs. Values are provided as means (a) and medians ± interquartile ranges (b).

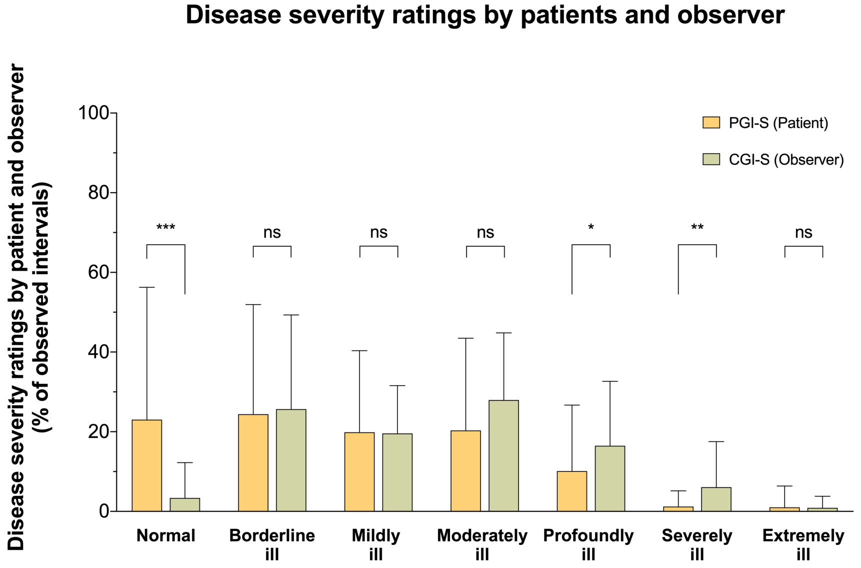

Supplementary Figure 4 | Perception of disease severity by patients and observer on the Patient Global Impression of Severity (PGI-S) and Clinical Global Impression of Severity (CGI-S). Supplementary Figure S4 shows the mean proportions of 1947 simultaneous half-hourly performed disease severity ratings from 51 patients with Parkinson's disease (yellow colour) and an independent clinical observer (green colour) using seven severity grades on the Patient Global Impression of Severity (PGI-S) and Clinical Global Impression of Severity (CGI-S), respectively. Values are means + standard deviations. *P<0.05, **P<0.01 and ***P<0.001 from Wilcoxon matched-pairs signed rank. ns: not significant.
